# Clear Cell Adenocarcinoma Arising from Abdominal Wall Endometriosis

**DOI:** 10.1155/2008/478325

**Published:** 2008-12-21

**Authors:** Thouraya Achach, Soumaya Rammeh, Amel Trabelsi, Rached Ltaief, Soumaya Ben Abdelkrim, Moncef Mokni, Sadok Korbi

**Affiliations:** ^1^Department of Pathology, University Teaching Hospital Farhat Hached, Sousse 4000, Tunisia; ^2^Department of Surgery, University Teaching Hospital Farhat Hached, Sousse 4000, Tunisia

## Abstract

Endometriosis is a frequent benign disorder. Malignancy arising in extraovarian endometriosis is a rare event. A 49-year-old woman is presented with a large painful abdominal wall mass. She underwent a myomectomy, 20 years before, for uterus leiomyoma. Computed tomography suggested that this was a desmoid tumor and she underwent surgery. Histological examination showed a clear cell adenocarcinoma associated with endometriosis foci. Pelvic ultrasound, computed tomography, and endometrial curettage did not show any malignancy or endometriosis in the uterus and ovaries. Adjuvant chemotherapy was recommended, but the patient was lost to follow up. Six months later, she returned with a recurrence of the abdominal wall mass. She was given chemotherapy and then she was reoperated.

## 1. Introduction

Endometriosis is a frequent benign disorder. Several observations of the coexistence of endometriosis and cancer have been published [1, 2]. Malignancy arising in extraovarian endometriosis is a rare event [1]. Here, we report a case of clear cell adenocarcinoma derived from pathologically confirmed endometriosis in the abdominal wall. We discuss the epidemiological and clinicopathological features of malignancy arising in abdominal wall endometriosis.

## 2. Case Report

**Figure 1:**
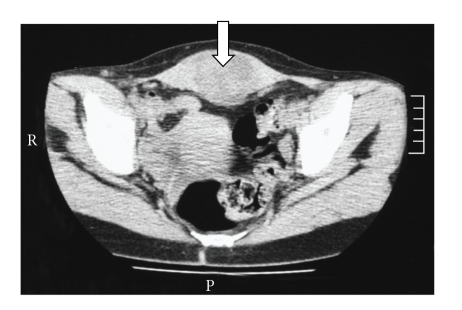
Computed tomograms of the abdomen show a heterogeneous and ill-defined tumor in the abdominal wall.

**Figure 2:**
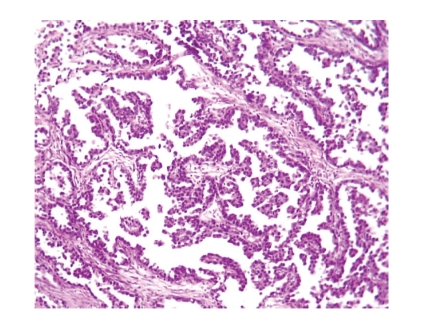
Tubulocystic and papillary pattern (hematoxylin eosin, original magnification ×200).

**Figure 3:**
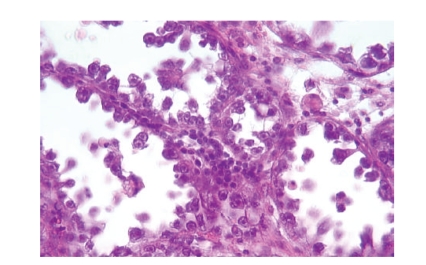
Tumor cells with hobnail configuration (hematoxylin eosin, original magnification ×400).

**Figure 4:**
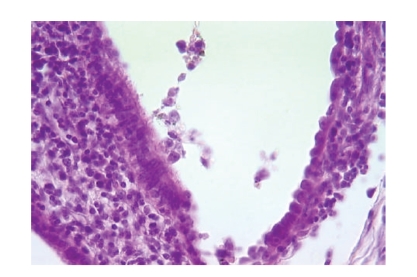
Endometrial gland invaded by tumor cells (hematoxylin eosin, original magnification ×400).

A 49 year-old woman, gravida 3, para 0, underwent a myomectomy, 20 years before, for uterus leiomyoma through a midline incision. She is presented with a painful large abdominal wall mass. On physical examination, a firm indurated mass was palpated in the lower abdominal wall. Abdominal and pelvic ultrasounds followed by computed tomography showed a heterogeneous intramuscular mass of 8.5 cm diameter without local extension (Figure 1). The clinical impression was of desmoid tumor and the patient taken to surgery and the tumor was resected without rupture. The surgeon did not perform any peritoneal washing or biopsies because of the absence of widespread tumor in the peritoneal cavity. The surgical specimen consisted of 11 cm cutaneous and muscular tissues, occupied by ill-defined white tumor, which contained cystic cavities and abundant foci of necrosis. Surgical margins were positive. Microscopically, the tumor showed a predominant papillary and tubulocystic growth pattern (Figure 2). The tumor cells were round or polygonal most with hobnail configuration (Figure 3). The cytoplasm was clear, and the nuclei were round with prominent nucleoli. Cellular atypia was moderate, and mitosis was rare. Benign endometriotic foci were observed in the proximity of the tumor (Figure 4). Immunohistochemically, tumor cells showed diffuse and strong cytoplasmic positivity with vimentin, epithelial membrane antigen, and cytokeratin 7, but no staining for cytokeratin 20 progesterone and estrogen receptor. Calretinin and mesothelin were negative. Pelvic ultrasound and computed tomography identified normal-sized ovaries and uterus. Endometrial curettage was negative for malignancy.

The diagnosis of clear cell adenocarcinoma arising from abdominal endometriosis foci was retained. Adjuvant chemotherapy was indicated, but the patient was lost to follow up. She returned six months after. At that time, ultrasound and computed tomography showed a recurrent mass at the abdominal wall with extension to the bladder. Three cycles of combination chemotherapy with cyclophosphamide and cisplatin were given, but the tumor did not regress. She underwent surgery again with a resection of 5 cm encapsulated nodule. At that time, uterus, ovaries, and tubes did not show any abnormalities. Histology demonstrated the same type of tumor. Margins were free of tumor. Three cycles of chemotherapy were also given but failed to control the disease; the chemotherapy she got is not precised. The computed tomography showed again a recurrent mass with extension to the bladder and pelvic bone, and adjuvant radiotherapy was indicated.

## 3. Discussion

Endometriosis, defined as the presence of endometrial-like tissue outside the uterine cavity, is usually located in the ovaries and pelvic peritoneum [2, 3]. Parietal endometriosis is very rare and constitutes 1 to 2% of endometriosis cases [1]. It arises usually in a surgical scar of cesarean section or hysterectomy, and less frequently in a surgical scar of hernia or of appendicectomy [1, 4]. Cases of endometriosis without scar have been described [1]. The incidence of abdominal wall endometriomas is of 0.04% among parturients undergoing cesarean section and it is more frequent than endometriosis following conventional gynaecologic surgery [4, 5]. In our case, abdominal wall endometriosis occurred in a surgical midline scar of myomectomy. The etiopathogenetic mechanism is more likely related to iatrogenic transplantation of endometrium during gynecological surgery rather than hematogenous dissemination or metaplasia [4, 6]. Clinical diagnosis remains difficult, and many patients are asymptomatic [4]. Symptoms related to pelvic endometriosis are noted in 26% of cases. Ultrasound can show a cystic lesion in many cases [5]. Women with pelvic endometriosis have a higher frequency of malignancy, but malignant change in extrapelvic endometriosis is a rare event [1, 2, 4, 7]. Twenty percent of malignancy in endometriosis occurs in extragonadal site [1, 8, 9]. There is extensive clinicopathological, molecular, and genetic evidence supporting the hypothesis that endometriosis is a neoplastic process with a potential for malignant transformation [3]. The natural course of malignant transformation of endometriosis is long and can be explained by estrogenic stimulation [8, 10–12]. Malignant transformation in endometriosis was first described by Sympson in 1925 in [9], who proposed three criteria for diagnosis: demonstration of a clear example of the endometriosis in proximity to the tumor, no other primary site for the tumor, and histologic appearance consistent with an origin from endometriosis. Scott in [1, 8] recommended the presence of transitional area between endometriosis and cancer. Atypical endometriosis, a term first coined by LaGrenade and Silvergerg in 1988 in [8, 13], is rare and is characterized by endometriotic glands with cytological and/or architectural atypia (hyperchromatic or pale nuclei with moderate to marked pleomorphism increased nuclear to cytoplasmic ratio, cellular crowding, stratification, or tufting). The rate of atypical endometriosis ranges from 1.7 to 3.6% in ovarian endometriosis [13]. Fukunaga et al. [13] demonstrated that atypical endometriosis in ovary is often associated with epithelial neoplasm and showed direct transition from atypical epithelium to malignant tumor. In our case, the criteria of Sympson were fully satisfied. The demonstration of endometriosis might require the examination of multiple levels and sections, that is why preoperative biopsy cannot make the diagnosis of malignancy arising in endometriosis [6]. Tumors that can arise in endometriosis include in decreasing order: endometrio id carcinoma (75.9–69.1%), sarcoma (25–11.6%), clear cell carcinoma (13.5–4.5%), and mucinous or serous carcinoma (4.6%–1%) [4]. In extrapelvic localization, clear cell carcinoma is the most common histological subtype, followed by endometrioid carcinoma [1]. Due to the rarity of malignant transformation of endometriosis at extragonadal sites, it is difficult to establish a treatment protocol. First-line treatment is surgery, removing as much endometriosis as possible, staging at this point is also necessary. Second-line treatment, with chemotherapy, radiotherapy, and even hormonotherapy may be needed. Prognosis is variable from 10 to 100% five-year survival, depending on histological type and localization of the disease [7].

## 4. Conclusion

Cutaneous localization of endometriosis is unusual and appears most frequently in surgical scars from obstetric or gynecological interventions. It is important to recognize the possibility of tumors arising from endometriosis when the pathologist is confronted to an extraovarian tumor with endometrial appearance. Examination of multiple sections is required to demonstrate endometriosis foci.
